# Knockout of *SlSBPASE* Suppresses Carbon Assimilation and Alters Nitrogen Metabolism in Tomato Plants

**DOI:** 10.3390/ijms19124046

**Published:** 2018-12-14

**Authors:** Fei Ding, Qiannan Hu, Meiling Wang, Shuoxin Zhang

**Affiliations:** College of Forestry, Northwest A&F University, Yangling 712100, Shaanxi, China; fding@nwafu.edu.cn (F.D.); anan1231993@sina.com (Q.H.); wangmeiling2081@163.com (M.W.)

**Keywords:** CRISPR/Cas9, SBPase, carbon assimilation, nitrogen metabolism, tomato

## Abstract

Sedoheptulose-1,7-bisphosphatase (SBPase) is an enzyme in the Calvin–Benson cycle and has been documented to be important in carbon assimilation, growth and stress tolerance in plants. However, information on the impact of SBPase on carbon assimilation and nitrogen metabolism in tomato plants (*Solanum lycopersicum*) is rather limited. In the present study, we investigated the role of SBPase in carbon assimilation and nitrogen metabolism in tomato plants by knocking out SBPase gene *SlSBPASE* using clustered regularly interspaced short palindromic repeats (CRISPR)/CRISPR-associated protein 9 (Cas9) gene editing technology. Compared with wild-type plants, *slsbpase* mutant plants displayed severe growth retardation. Further analyses showed that knockout of *SlSBPASE* led to a substantial reduction in SBPase activity and as a consequence, ribulose-1,5-bisphosphate (RuBP) regeneration and carbon assimilation rate were dramatically inhibited in *slsbpase* mutant plants. It was further observed that much lower levels of sucrose and starch were accumulated in *slsbpase* mutant plants than their wild-type counterparts during the photoperiod. Intriguingly, mutation in *SlSBPASE* altered nitrogen metabolism as demonstrated by changes in levels of protein and amino acids and activities of nitrogen metabolic enzymes. Collectively, our data suggest that *SlSBPASE* is required for optimal growth, carbon assimilation and nitrogen metabolism in tomato plants.

## 1. Introduction

The Calvin–Benson cycle is the primary pathway of photosynthetic carbon fixation in higher plants. The cycle is central to plant metabolism, providing intermediates for biosynthesis of sucrose, starch, isoprenoid and shikimic acid [1]. Within the cycle, there are a total of 11 different enzymes that catalyze 13 reactions, which are divided into three distinct phases, including carboxylation of ribulose-1,5-bisphosphate (RuBP), reduction of 3-phosphoglycerate and regeneration of the CO_2_ acceptor RuBP [2]. The enzyme sedoheptulose-1,7-bisphosphatase (SBPase) catalyzes the dephosphorylation of sedoheptulose-1,7-bisphosphate to sedoheptulose-7-phosphate, acting in the regeneration of CO_2_ acceptor molecule RuBP in the Calvin–Benson cycle. In vitro studies have revealed that the activity of SBPase is regulated by a variety of factors, including pH and Mg^2+^ [3,4]. In addition, like other enzymes in the Calvin-Benson cycle, SBPase is activated via ferredoxin/thioredoxin system in response to light [5,6].

Multiple lines of evidence support that SBPase is a critical enzyme in the regulation of photosynthetic carbon fixation in the Calvin-Benson cycle and is therefore a target to manipulate to improve photosynthetic capacity in plants. In the early work, expression of cyanobacterial FBPase (fructose-1,6-bisphosatase) /SBPase in tobacco plants led to enhanced photosynthetic carbon fixation and biomass [7]. In another study, activity of SBPase was increased by overexpression of an *Arabidopsis* cDNA in tobacco plants and higher levels of carbohydrates accumulation and biomass were observed in transgenic plants [8]. Later work has shown that overexpression of SBPase improved photosynthetic carbon gain and yield in tobacco plants grown under field conditions [9]. Additionally, analysis of a loss-of-function mutant illustrated that mutation in *SBPASE* retarded growth and development through inhibition of carbon assimilation in *Arabidopsis* [10]. More recently, expression of a *Brachypodium distachyon* SBPase gene in wheat increased SBPase activity and improved photosynthesis and grain yield [11].

Nitrogen is an essential nutrient element for plant growth and development. It is well known that nitrogen is a major constituent of numerous organic compounds, including nucleic acids, amino acids, chlorophyll and phytohormones. Nitrogen assimilation represents a crucial step in the synthesis of organic compounds that require nitrogen and this step involves several enzymes, including nitrate reductase (NR), glutamine synthetase (GS), glutamate synthase (GOGAT) and glutamate dehydrogenase (GDH) [12,13]. It is recognized that nitrogen assimilation is in close association with carbon metabolism and the balance between nitrogen and carbon metabolism is critical for optimal growth and development in plants [14,15]. Nitrate availability affects the expression of a variety of genes encoding enzymes in carbon metabolism, while carbon depletion decreases protein biosynthesis and alters nitrogen metabolism [14,16]. A previous study has concluded that reduced SBPase activity in transgenic rice leads to decreased nitrogen use efficiency [17], however, no further studies have been conducted to investigate the impact of SBPase activity on nitrogen metabolism. Studies using *SBPASE* knockout mutant may help to gain further insights into the relationship between SBPase activity and nitrogen metabolism in plants.

Genome editing holds great promise for crop improvement. The clustered regularly interspaced short palindromic repeats (CRISPR)/CRISPR-associated protein 9 (CRISPR/Cas9) system is a powerful tool for genome editing. Since the first report of CRISPR/Cas9 editing in plants in 2013, this system has been widely used for genome editing in model plants and crops, including *Arabidopsis thaliana*, rice, wheat, maize and tomato [18,19,20,21]. CRISPR/Cas9 has become the tool of choice for gene editing due to several advantages, such as high mutation efficiency, rare off-target mutations, stable inheritance, low cost and great flexibility [22]. Tomato (*Solanum lycopersicum*) is a high-value vegetable crop, making an important part of agricultural industry. Using antisense transgenic plants, we have previously investigated the importance of tomato SBPase gene *SlSBPASE* (Solyc05g052600) in the photosynthetic carbon assimilation and growth in tomato plants [23]. However, studies using partial-loss/gain-of-function transgenic plants may yield conflicting results due to the inconsistent expression of target genes within or among generations. In addition, the absence of *SlSBPASE* knockout mutant hampers us from making a thorough functional analysis of this gene. In the present study, CRISPR/Cas9 system was utilized to generate *SlSBPASE* knockout mutant plants, which allowed us to better define the functions of SBPase in tomato plants. We were in an attempt to investigate the role for SBPase in the regulation of carbon assimilation and nitrogen metabolism by measuring photosynthetic carbon fixation, carbohydrate accumulation, protein content and enzyme activities related to nitrogen metabolism in *slsbpase* mutant plants and their wild-type counterparts.

## 2. Results

### 2.1. Generation of Loss-of-Function Mutant of SlSBPASE Using CRISPR/Cas9 Gene-Editing System

**Figure 1:**
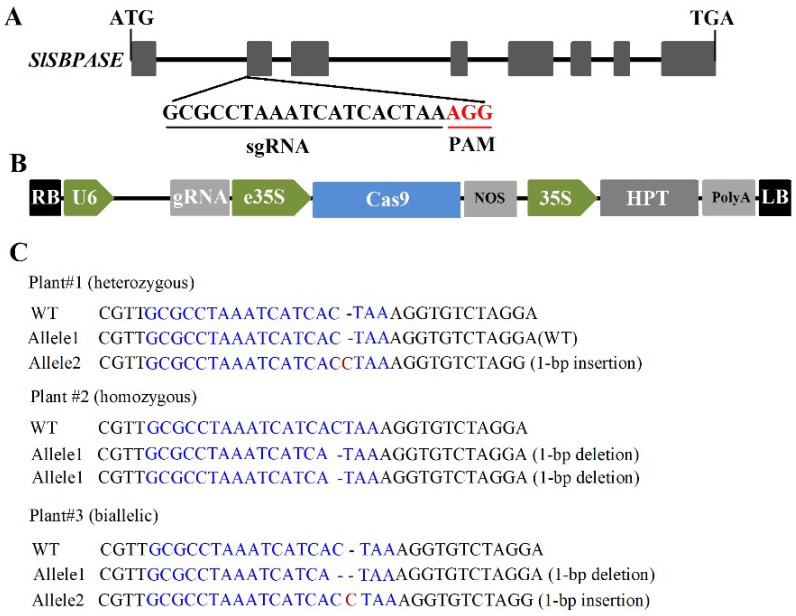
Schematic diagram of *SlSBPASE* gene structure, vector structure and CRSIPR/Cas9-induced mutagenesis. (**A**) Schematic diagram of *SlSBPASE* gene structure and target sequence. ATG, start codon; TGA, stop codon; sgRNA, single guide RNA; PAM, protospacer adjacent motif; AGG in red, PAM sequence. (**B**) Schematic diagram of the vector used in this study. LB, left border of T-DNA (Transfer DNA); RB, right border of T-DNA; U6, Arobidopsis U6 promoter; gRNA, guide RNA; e35S, enhanced 35S promoter; Cas9, optimized Cas9; NOS (nopaline synthase) Ter, NOS terminator; 35S, CaMV (Cauliflower mosaic virus) 35S promoter; HPT (hygromycin phosphotransferase), hygromycin selection marker; PolyA Ter, PolyA terminator. (**C**) Representative genotypes of *slsbpase* mutants. WT, wild type. Target sequences are in blue.

SBPase acts in the regenerative phase of the Calvin–Benson cycle and is important in the control of carbon flux in plants. Our previous studies on SBPase using either SBPase overexpressing or antisense transgenic tomato plants have exhibited a critical role of SBPase in photosynthetic carbon assimilation [23,24]. In tomato plants, SBPase is encoded by one gene *SlSBPASE*, the transcript of which is most abundant in leaf [23]. In order to provide further evidence for the role of SBPase in the regulation of carbon fixation, we generated stable loss-of-function *slspbase* mutants using CRISPR/Cas9 gene-editing system. A single guide RNA (sgRNA, 5′-GCGCCTAAATCATCACTAA-3′) was designed to specifically target the second exon of *SlSBPASE* (Figure 1A). We cloned the sgRNA sequence into a binary vector that contains sgRNA and Cas9 expression cassettes and the resulting construct was transformed into wild-type tomato plants. 

T0 transgenic plants were genotyped by sequencing PCR products from genomic DNA flanking the target site and mutant plants were confirmed. In these mutant plants, biallelic, heterozygous and homozygous mutations were found and two of the plants carried the same homozygous 1-bp deletion in the second exon of *SlSBPASE* (Figure 1C). Homozygous T1 plants were selected for further analyses in this study and no off-target mutations in these plants were found in the three predicted off-target sites.

### 2.2. Characterization of slsbpase Mutant Plants

**Figure 2:**
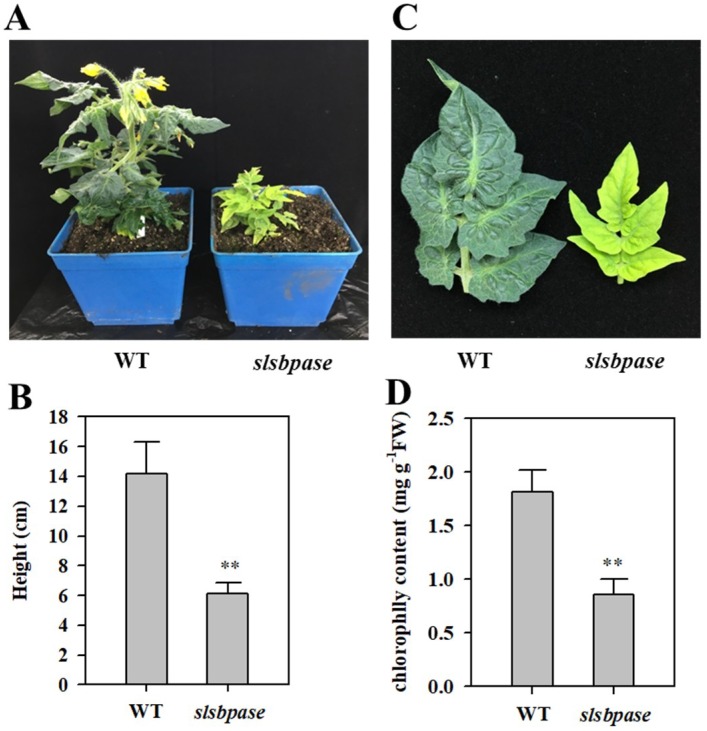
Characterization of *slsbpase* mutant. (**A**) Phenotypes of *slsbpase* mutant plants (*slsbpase*) and wild-type plants (WT). (**B**) Plant height of wild-type and mutant plants. (**C**) Leaf chlorosis phenotype of *slsbpase* mutant. (**D**) Leaf chlorophyll content of wild-type and mutant plants. The values presented are means ± SDs (standard deviation, *n* = 3). Asterisks indicate significant difference at ** *p* < 0.01 between *slsbpase* mutant plants and wild-type plants.

Growth of *slsbpase* mutant plants was dramatically inhibited compared with that of wild-type plants at both vegetative and reproductive stages. The height and the number of leaves were substantially reduced by mutation of *SlSBPASE* (Figure 2A,B). The number of flowers in *slsbpase* mutant plants was also decreased. Moreover, *slsbpase* mutants exhibited a leaf chlorotic phenotype (Figure 2C), with the content of chlorophyll being substantially reduced (Figure 2D). In spite of severe retardation of growth, *slsbpase* mutants were still able to flower and set fruits under optimal growth conditions.

### 2.3. Mutation of SlSBPASE Causes Severe Reductions in SBPase Activity, RuBP Regeneration and CO_2_ Assimilation Rate

**Figure 3:**
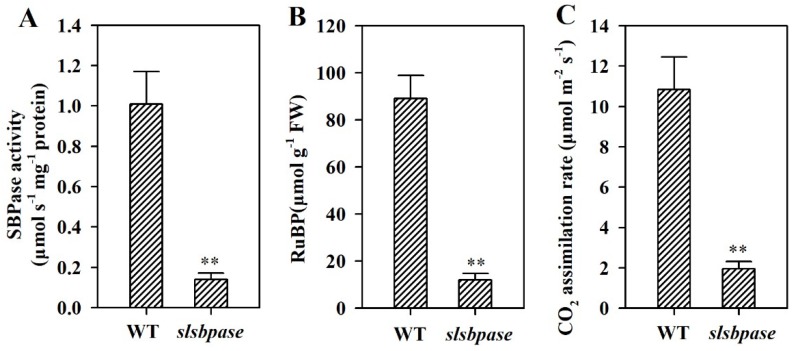
Changes of SBPase activity (**A**), RuBP regeneration (**B**) and CO_2_ assimilation rates (**C**) in *slsbpase* mutant plants. The values presented are means ± SDs (*n* = 3). Asterisks indicate significant difference at ** *p* < 0.01 between *slsbpase* mutant plants and wild-type plants.

SBPase is thought to be responsible for the regeneration of RuBP and affects the photosynthetic carbon fixation [25]. To validate the relationship between mutation of *SlSBPASE* and reductions in SBPase activity and CO_2_ assimilation rate, we first measured SBPase activity of *slsbpase* mutants. A low level of SBPase activity was detected in the leaves of mutant plants. SBPase activity in *slsbpase* mutant leaves was reduced to 11% of that in wild-type leaves (Figure 3A). Given that *SlSBPASE* was mutated, this result came as unexpected. It is likely that this small amount of activity was caused by non-specific activity of other proteins in the measurement of SBPase activity. Similar result of trace SBPase activity was also observed in an *Arabidopsis* mutant with loss of function of *SBPASE* [10].

SBPase functions in the regenerative stage and is crucial for the regeneration of CO_2_ acceptor molecule RuBP in the Calvin–Benson cycle. We therefore determined the accumulation of RuBP in *slsbpase* mutants to examine the effect of decreased SBPase activity on the regeneration of RuBP. In this study, it was observed that RuBP accumulation in *slsbpase* mutant plants was dramatically reduced compared with that in wild-type plants (Figure 3B), suggestive of the key role for SBPase in controlling RuBP regeneration.

To investigate the role of SBPase in carbon assimilation, we then measured CO_2_ assimilation rate of *slsbpase* mutants. It was found that CO_2_ assimilation was substantially inhibited, with carbon assimilation rate in *slsbpase* mutant being reduced to 18% of that in their wild-type counterparts (Figure 3C). This observation is consistent with a previous report that decreases in SBPase activity result in significant reductions in photosynthesis in transgenic tobacco plants [26,27].

### 2.4. Mutation of SlSBPASE Dramatically Inhibits Biosynthesis of Sucrose and Starch

**Figure 4:**
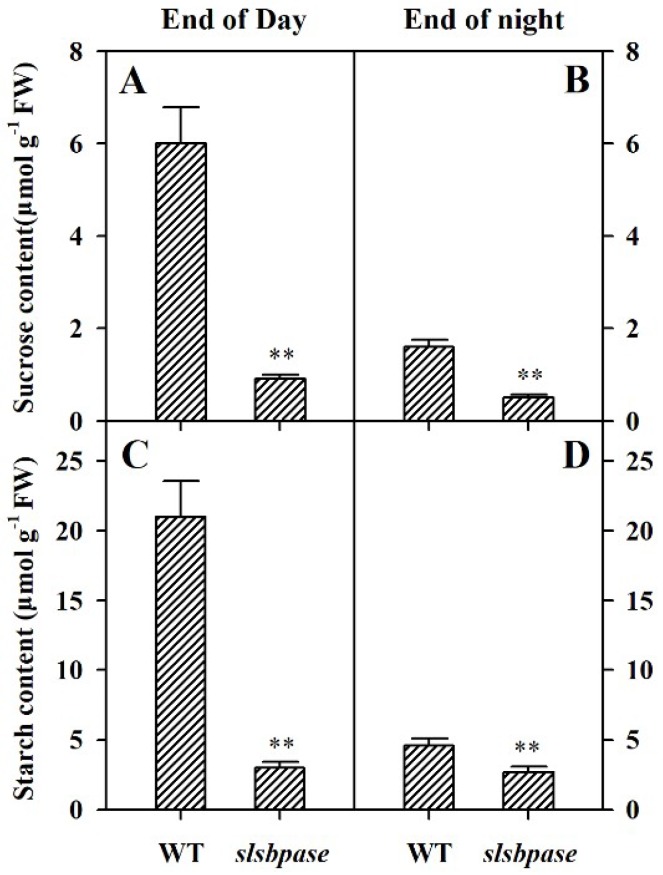
Diurnal carbohydrate accumulation in wild-type and *slsbpase* mutant plants. Levels of sucrose (**A**,**B**) and starch (**C**,**D**) were determined in leaves of wild-type and *slsbpase* mutant plants in the light period (end of day) and at the end of the night. The values presented are means ± SDs (*n* = 3). Asterisks indicate significant difference at ** *p* < 0.01 between *slsbpase* mutant plants and wild-type plants.

To examine the effect of the *SlSBPASE* mutation on the accumulation of carbohydrates in *slsbpase* mutant plants, in parallel with photosynthetic analysis, we measured the contents of sucrose and starch in the mutant and wild-type plants at the end of the light period and at the end of dark period (16-h light/8-h dark). In both the mutant plants and their wild-type counterparts, sucrose and starch accumulated during the light period. However, the mutant plants accumulated substantially less sucrose and starch than wild-type plants (Figure 4A,C), in agreement with the role of SBPase in photosynthetic carbon fixation. At the end of dark period, levels of sucrose and starch were low in both *slsbpase* mutant and wild-type plants (Figure 4B,D), demonstrating that the carbohydrates accumulated in the light were being remobilized at night.

### 2.5. Mutation of SlSBPASE Reduces Night Respiration Rate

**Figure 5:**
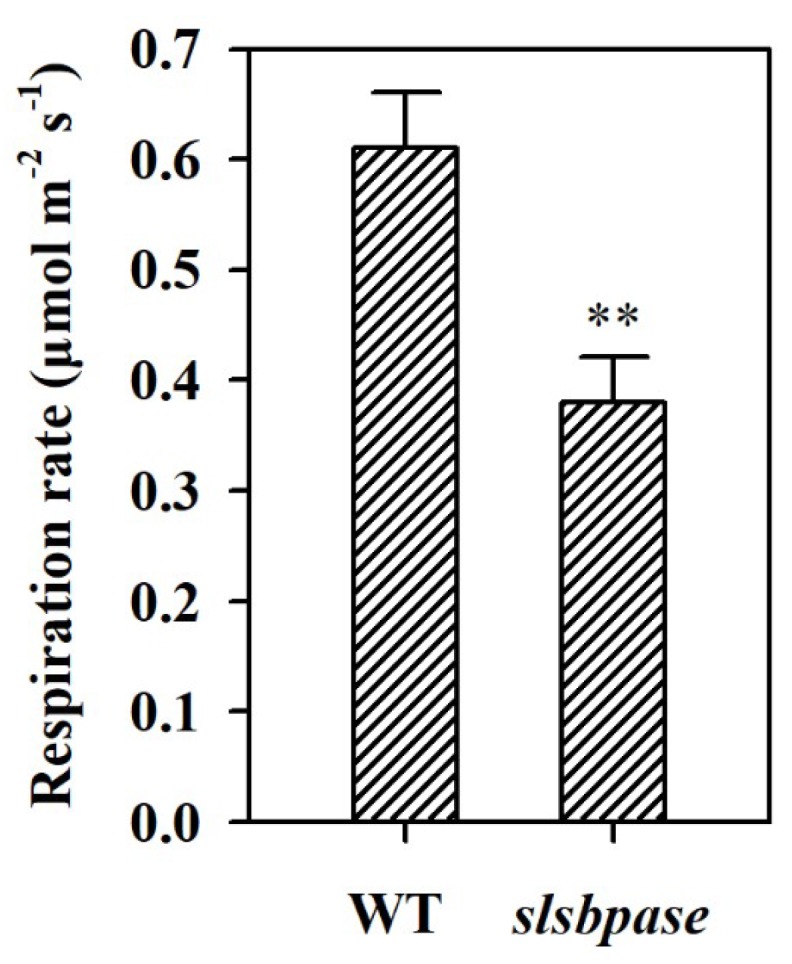
Night respiration rates in wild-type tomato plants and *slsbpase* mutant plants. The values presented are means ± SDs (*n* = 3). Asterisks indicate significant difference at ** *p* < 0.01 between *slsbpase* mutant plants and wild-type plants.

In order to further understand the difference in the accumulation of starch between wild-type and *slsbpase* mutant plants, we measured the night respiration rate. It was observed that respiration rate was decreased in *slsbpase* mutant plants at night compared with that in wild-type plants (Figure 5). This result was consistent with our observation that starch was reduced by ~10% in *slsbpase* mutant plants, while starch was decreased by ~78% in wild-type plants at the end of night. 

### 2.6. Mutation of SlSBPASE Decreases Levels of Protein and Amino Acids in Tomato Leaves

**Figure 6:**
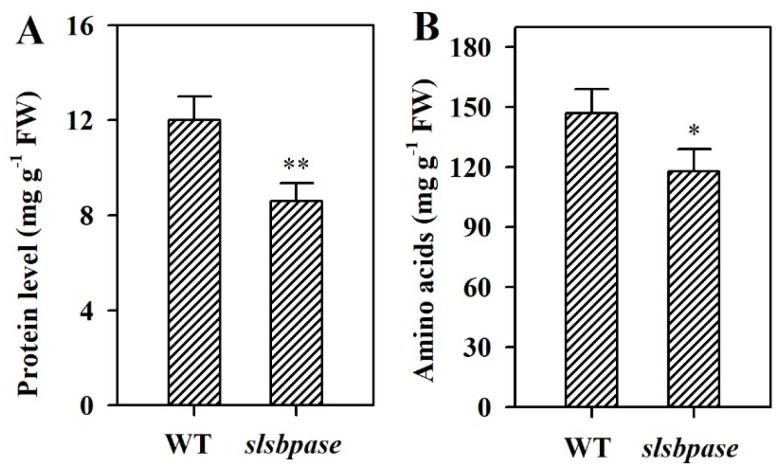
Changes in levels of total protein and amino acids as affected by mutation of *SlSBPASE* in tomato plants. (**A**) Protein level. (**B**) Amino acids level. The values presented are means ± SDs (*n* = 3). Asterisks indicate significant difference at ** *p* < 0.01 and * *p* < 0.05 between *slsbpase* mutant plants and wild-type plants.

To examine the impact of *SlSBPASE* mutation on nitrogen metabolism, we measured the contents of protein and amino acids in *slsbpase* mutant plants and their wild-type counterparts. In *slsbpase* mutant leaves, total protein was decreased by ~30% compared with wild-type leaves (Figure 6A). Similar to protein level, level of amino acids was reduced as a consequence of *SlSBPASE* mutation. Level of amino acids was reduced by ~14% in *slsbpase* mutant plants compared with their wild-type counterparts (Figure 6B). The decreased levels of protein and amino acids suggest that mutation in *SlSBPASE* leads to alterations in nitrogen metabolism.

### 2.7. Mutation of SlSBPASE Alters Activities of Enzymes Involved in Nitrogen Metabolism

**Figure 7:**
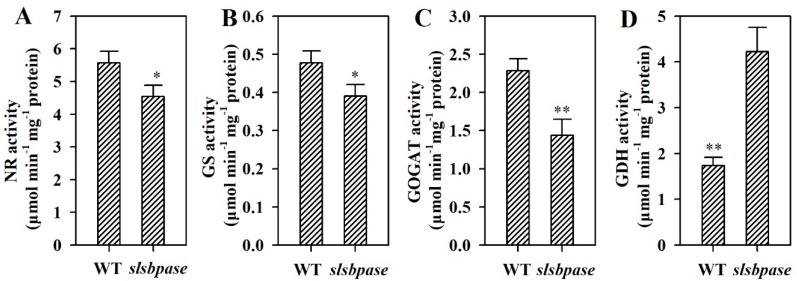
Activities of metabolic enzymes involved in nitrogen metabolism in wild-type plants and *slsbpase* mutant plants. (**A**) NR. (**B**) GS. (**C**) GOGAT. (**D**) GDH. The values presented are means ± SDs (*n* = 3). Asterisks indicate significant difference at ** *p* < 0.01 and * *p* < 0.05 between *slsbpase* mutant plants and wild-type plants.

To gain further insights into the impact of mutagenesis of SBPase gene on nitrogen metabolism, we measured activities of nitrate reductase (NR), glutamine synthetase (GS), glutamate synthase (GOGAT) and glutamate dehydrogenase (GDH), which are key enzymes in the nitrogen metabolism. Mutation in *SlSBPASE* led to reductions in the activities of NR, GS and GOGAT in tomato leaves (Figure 7A–C). Contrary to NR, GS and GOGAT, GDH activity was significantly enhanced in *slsbpase* mutant leaves compared with wild-type leaves (Figure 7D).

## 3. Discussion

SBPase has been demonstrated to be an important enzyme mediating the carbon flux in the Calvin–Benson cycle. Previous studies have produced transgenic plants displaying a range of SBPase activities to investigate the role of SBPase in photosynthetic capacity, growth and tolerance to abiotic stresses [8,11,23,24,26,28,29,30,31,32]. In this study, we generated loss-of-function mutants of *SlSBPASE* using CRISPR/Cas9 gene-editing system in tomato plants to confirm the function of SBPase in photosynthetic carbon assimilation and growth and to examine the impact of *SlSBPASE* mutation on nitrogen metabolism. We concluded that SBPase plays a critical role in growth and carbon assimilation and suppression of SBPase activity alters nitrogen metabolism in tomato plants. The evidence supporting this conclusion includes that (1) CRISPR/Cas9-mediated mutagenesis of *SlSBPASE* led to the phenotype of dramatic growth retardation; (2) *slsbpase* mutant plants exhibited a substantial reduction in SBPase activity, RuBP regeneration and carbon assimilation rate; (3) mutation of *SlSBPASE* decreased the accumulation of carbohydrates; (4) mutation of *SlSBPASE* altered levels of protein and amino acids and activities of key metabolic enzymes in nitrogen metabolism.

The Calvin–Benson cycle is the primary photosynthetic carbon assimilation pathway and can be limited by several enzymes, including Rubisco, fructose-1,6-bisphosatase (FBPase), SBPase and phosphoribulokinase (PRKase). SBPase functions in the regenerative phase of the Calvin–Benson Cycle, catalyzing the irreversible dephosphorylation of sedoheptulose-1,7-biphosphate to sedoheptulose-7-phosphate. SBPase has been proved to exert control over flux of carbon through the cycle. Given the importance of SBPase, it has been extensively studied using transgenic lines of both model and crop plants. In tobacco plants with decreased SBPase activity, photosynthetic capacity and growth were reduced [25,28]. On the contrary, elevated levels of SBPase resulted in increased carbon assimilation rates and biomass in tobacco plants [8,9]. These results were also observed in transgenic crops with different levels of SBPase, such as tomato, rice and wheat [11,17,23,30]. To provide additional and direct evidence for the role of SBPase, in this work, we generated loss-of-function mutants of *SlSBPASE* using CRISPR/Cas9 gene-editing system, which has proved powerful and efficient in knocking out genes of interest. The *slsbpase* mutant plants displayed the phenotype of severe growth retardation, with plant size being reduced and the number of leaves decreased, supporting the notion that *SlSBPASE* is required for normal growth in tomato plants. 

It was observed that *slsbpase* mutant plants had a chlorotic leaf phenotype. Similar phenotype was also observed in *sbpase* mutant plants of Arabidopsis [10]. One possible explanation of leaf chlorosis is that *SlSBPASE* mutation led to the inhibition of chloroplast biogenesis, which may result from the limited carbon reserve such as starch. The other explanation is that mutation in *SlSBPASE* might accelerate the degradation of chlorophyll as loss of *SlSBPASE* was previously demonstrated to trigger premature leaf senescence [33]. Typically, degradation of chlorophyll was closely associated with leaf senescence.

SBPase plays an essential role in the regeneration of RuBP, which is the CO_2_ acceptor and, in association with Rubisco activase, is crucial for carbon assimilation by affecting the activation state of Rubisco [34]. The regenerative capacity for RuBP in the Calvin-Benson cycle is largely dependent on SBPase activity. It has been illustrated that RuBP regeneration decreases linearly with reduced SBPase activity in tobacco plants [25]. Consistently, in this study, we observed that mutation of *SlSBPASE* led to severe reduction in SBPase activity, which consequently decreased RuBP accumulation and carbon assimilation rate. This observation supports the critical of SBPase in retaining photosynthetic carbon assimilation in tomato plants. 

Though growth was severely retarded, *slsbpase* mutant plants were still able to survive and produce seeds. Given that RuBP regeneration is indispensable for carbon fixation, there may be an enzyme that possesses catalytic activity of SBPase, catalyzing the dephosphorylation of sedoheptulose-1,7-biphosphate to sedoheptulose-7-phosphate at a very low efficiency in *slsbpase* mutant plants. Chloroplast FBPase might be a candidate enzyme because FBPase and SBPase have similar architecture and their regulatory and catalytic properties resemble. In addition, there are also some amino acids that are conserved in the substrate binding domain of both enzymes [35]. However, it must be noted that the conclusion from a recent study refutes FBPase as a possible substitute. Gütle et al. claimed that SBPase has a larger substrate binding site than FBPase, accommodating both seven- and six-carbon sugar phosphate substrates, while FBPase is active only with six-carbon substrates [36]. In this scenario, there may exist a third enzyme for the dephosphorylation of sedoheptulose-1,7-biphosphate to sedoheptulose-7-phosphate operating inefficiently. It is also possible that an unknown mechanism is present in plants, which might bypass the pathway of sedoheptulose-1,7-biphosphate and aids in the minimum regeneration of RuBP to guarantee the survival of plants in the absence of enough SBPase. 

The rate of plant growth partly depends on the efficiency of photosynthetic carbon assimilation. In the present work, the retarded growth was observed concurrent with suppressed carbon assimilation rate in *SlSBPASE*-knockout plants. Analysis of carbohydrate accumulation during the light period showed that levels of sucrose and starch were much reduced in *slsbpase* mutant plants in comparison with those in wild-type plants. Thus, there was evident correlation between retarded growth, reduced SBPase activity, suppressed photosynthesis and decreased accumulation of sucrose and starch. However, at the end of dark period, accumulation of carbohydrate was reduced to a low level in wild-type plants, suggesting carbohydrates produced in the light period were being used possibly for growth in the dark period. As such, the difference in the consumption of carbohydrates in the dark between mutant and wild-type plants may explain, in part, the growth difference between them.

It has been established that carbon metabolism and nitrogen metabolism are interrelated in plants [14,15,16]. In this study, we found that mutation in *SlSBPASE* severely reduced SBPase activity, leading to suppressed carbon assimilation, which further altered nitrogen metabolism, as demonstrated by reduced levels of proteins and amino acids and altered activities of nitrogen metabolic enzymes in *slsbpase* mutant plants. The enzymes NR, GS and GOGAT are required for the assimilation of inorganic nitrogen. It was observed that the activities of these enzymes were markedly decreased by mutation of *SlSBPASE*, implying that nitrogen assimilation was reduced as a consequence of suppressed SBPase. Nitrogen assimilation is among the most energy-intensive reactions. It is estimated that the energy needed for assimilating each NO^3−^ is equivalent to 12 ATP (adenosine triphosphate), while most biochemical reactions consume the energy of one or two ATP [37]. We thus reasoned that downregulation of nitrogen assimilation may be a general response to reduced energy reserve in *slsbpase* mutant plants. GDH (glutamate dehydrogenase) has been considered as a metabolic indicator of carbon deficiency [16]. During carbon shortage, GDH acts in the direction of releasing amino nitrogen and recycles glutamate to 2-oxoglutarate for the tricarboxylic acid (TCA) cycle [38,39]. Consistently, we observed that in contrast to NR, GS and GOGAT, GDH activity was significantly increased by knockout of *SlSBPASE*, demonstrating the increased nitrogen catabolism in *slsbpase* mutant plants. These results support that *slsbpase* mutant plants are able to adjust central nitrogen metabolism in response to carbon deficiency caused by loss of SBPase activity.

In summary, we generated a loss-of-function mutant of *SlSBPASE* using CRISPR/Cas9 mediated gene-editing system. We have demonstrated that *SlSBPASE* is required for optimal growth, carbon assimilation and nitrogen metabolism in tomato plants. Mutation in *SlSBPASE* leads to reduced photosynthesis, decreased carbohydrate accumulation, altered nitrogen metabolism and retarded growth in *slsbpase* mutant plants. Our study provides evidence that SBPase, as an individual enzyme in the Calvin–Benson cycle, not only exerts control over carbon assimilation but also impacts nitrogen metabolism in plants.

## 4. Materials and Methods

### 4.1. Plant Materials

Tomato (*Solanum lycopersicum* cv. Micro-Tom) *slsbpase* mutant and wild-type seeds were sterilized and germinated at 25 °C in the dark on filter paper in petri dishes. Germinated seeds were then planted individually in 12 cm × 12 cm × 10 cm plastic pots filled with peat and vermiculite (3/1 *v*/*v*). Plants were grown under following conditions: 380 μmol mol^−1^ of CO_2_, photon flux density of 300 μmol m^−2^s^−1^, day/night temperature of 25/20 °C, relative humidity of 60% and a photoperiod of 16 h.

### 4.2. Selection of Target Sequence and Plasmid Construction

A target sequence (5′-TGCGCCTAAATCATCACTAAAGG-3′) in the second exon of *SLSBPASE* (Solyc05g052600) was selected using CRISPR online tool (available online: http://skl.scau.edu.cn/targetdesign/) [40]. The CRISPR/Cas9-*SlSBPASE* vector was constructed using a kit (BGK01) according to the manufacturer’s instructions (BIOGLE, Changzhou, Jiangsu, China). A 19-bp sgRNA oligo (5′-GCGCCTAAATCATCACTAA-3′) was introduced into the expression cassette, including chimeric RNA driven by the AtU6 promoter and optimized Cas9 driven by the enhanced CaMV 35S promoter.

### 4.3. Agrobacterium Tumefaciens-Mediated Transformation

The CRISPR/Cas9-*SlSBPASE*-expressing plasmid was introduced into *Agrobacterium tumefaciens* strain EHA105 (Waryong, Beijing, China). Transgenic tomato plants were produced using the *Agrobacterium tumefaciens*-mediated transformation method [41]. Transgenic plants were selected by hygromycin resistance and were maintained in a growth chamber at 25 °C with a photoperiod of 16 h light and 8 h dark.

### 4.4. Mutation Analysis of Transgenic Lines

The genomic DNA was extracted from leaves of transgenic plants using the DNeasy^®^ Plant Mini Kit (TIANGEN Biotech Co. Ltd., Beijing, China) and was used as template to amplify *SlSBPASE* fragment using primers ATGGATTTGGCATAGCCTAGTAGCT (Forward) and ATCATTAACCTGATTAACCCCTTAT (Reverse) by PCR. The PCR products were sequenced and the sequencing chromatograms were analyzed using a web-based tool DSDecode (available online: http://skl.scau.edu.cn/dsdecode/) [40].

### 4.5. Off-Target Analysis

Potential off-target sites were predicted using a web-based tool (available online: http://skl.scau.edu.cn/offtarget/) [40]. Three potential off-target sites were selected (Table 1). The genomic DNA was extracted from leaves of T1 transgenic tomato plants and was used to amplify fragments surrounding the potential off-target sites using specific primers (Table 1). The PCR products were sequenced and analyzed.

### 4.6. Activities of SBPase, NR, GS, GOGAT and GDH

SBPase activity was measured according to the protocol of Harrison et al. [26]. Leaf samples of mutant and wild-type plants were thoroughly ground in liquid nitrogen and then transferred to 1 mL extraction buffer containing 50 mM Hepes, pH 8.2; 5 mM MgCl_2_; 1 mM EDTA (ethylenediaminetetraacetic acid); 1 mM EGTA (ethylene glycol-bis(β-aminoethyl ether)-N,N,N′,N′-tetraacetic acid); 10% glycerol; 2 mM benzamidine; 2 mM amino caproic acid; 0.5 mM phenylmethylsulfonyluoride (PMSF); 10 mM dithiothreitol (DTT). The supernatant after centrifuging was collected and passed through a NAP-10 column (GE Healthcare Life Sciences, Pittsburgh, PA, USA) equilibrated with desalting buffer. For the assay, 20 μL of protein samples was added to 80 μL of assay buffer (50 mM Tris, pH 8.2; 15 mM MgCl_2_; 1.5 mM EDTA; 10 mM DTT; 2 mM sedoheptulose-7-phosphate (SBP) and incubated at 25 °C for 5 min. The reaction was stopped by adding 50 μL of 1 M perchloric acid. The samples were then centrifuged for 5 min and the supernatant assayed for phosphate. Fifty microliter samples and phosphate standards (0–0.5 mM NaH_2_PO_4_) were incubated with 850 μL molybdate solution (0.3% ammonium molybdate in 0.55 M H_2_SO_4_) for 10 min at room temperature. Malachite green (0.035% malachite green, 0.35% polyvinyl alcohol) was added (150 μL) and the samples incubated for a further 45 min at room temperature. Absorbance at 620 nm was measured and used for calculation of SBPase activity.

Nitrate reductase (NR) was extracted and measured using a Nitrate Reductase (NR) Assay Kit (BC0080, Solarbio, Beijing, China). Briefly, 0.1 g leaf samples were extracted in 1 ml extraction solution and the mixture was centrifuged at 4000 *g* for 10 min. The resulting supernatant was collected for further analysis. The absorbance at 520 nm was used for the calculation of NR activity.

Glutamine synthetase (GS) was extracted and measured using a Micro Glutamine Synthetase (GS) Assay Kit (BC0915, Solarbio, Beijing, China). Briefly, 0.1 g leaf samples were thoroughly ground in liquid nitrogen and extracted with 1 mL extraction buffer. The mixture was centrifuged at 8000 *g* at 4 °C for 10 min. The supernatant after centrifuging was collected for activity measurement. The absorbance at 520 nm was used for the calculation of GS activity.

Glutamate synthase was extracted and measured using a Glutamate Synthase (GOGAT) Assay Kit (BC0070, Solarbio, Beijing, China). Briefly, 0.1 g leaf samples were extracted in 1 mL extraction buffer. The extraction mixture was centrifuged at 10,000 *g* at 4 °C for 10 min. The resulting supernatant was harvested and the absorbance at 340 nm was measured for the calculation of GOGAT activity.

Glutamate dehydrogenase Glutamate Dehydrogenase (GDH) Assay Kit (BC1460, Solarbio, Beijing, China). In brief, 0.1 g leaf samples were extracted in 1 mL GDH extraction buffer. The extraction mixture was centrifuged at 8000 *g* at 4 °C for 10 min. The resulting supernatant was collected for further analysis. The absorbance at 340 nm was measured for the calculation of GDH activity.

### 4.7. Measurement of Carbon Assimilation Rate and Night Respiration Rate

Carbon assimilation rate and night respiration rate were measured with a photosynthesis system LI-6400XT (LI-COR Biosciences, Lincoln, NE, USA). The measurements were made on young fully expanded leaves of *slsbpase* and wild-type plants.

### 4.8. Determination of RuBP Level

Fresh leaf samples (0.1 g) were ground with 1 ml of 5% HClO_4_ and the level of phosphorylated RuBP was determined enzymatically as described by Sicher et al. [42].

### 4.9. Determination of Carbohydrate Level

Same leaves used for measurements of photosynthesis in mutant and wild-type tomato plants were harvested at the end/beginning of day for carbohydrate analysis. The carbohydrate levels were determined as described by Maeda et al. and Stitt et al. [43,44].

### 4.10. Measurements of Protein and Amino Acids

Leaf samples were collected from slsbpase mutant plants and wild-type plants. Total protein was extracted using a Plant Total Protein Extraction Kit (Solarbio, Beijing, China) and quantified by Bradford assay [45]. Amino acids were extracted and measured using a kit (BC1575, Solarbio, Beijing, China)

### 4.11. Statistical Analysis

Experiments in this study were repeated three times and the values presented are the means ± SDs. Student’s *t* test was performed to compare the difference between wild-type plants and mutant plants. Asterisks indicate that mean values are significantly different at *p* < 0.05 or *p* < 0.01 between wild-type and *slsbpase* mutant plants.

## Figures and Tables

**Table 1:** Potential off-target sites and primers used in this study.

Potential off-Target Sites	Primer Sequence (5′–3′)
Site 1	F1: TGGGTCTGAATCATCACTAG
TGGGTCTGAATCATCACTAGAGG	R1: CTAGTGATGATTCAGACCCA
Site 2	F2: AGTGCCTAAATCCTCAATAA
AGTGCCTAAATCCTCAATAAAGG	R2: TTATTGAGGATTTAGGCACT
Site 3	F3: TGTGTCTAAATCATCATTCA
TGTGTCTAAATCATCATTCAAGG	R3: TGAATGATGATTTAGACACA
